# Media intervention program for reducing unrealistic optimism bias: The link between unrealistic optimism, well‐being, and health

**DOI:** 10.1111/aphw.12316

**Published:** 2021-10-24

**Authors:** Dariusz Dolinski, Wojciech Kulesza, Paweł Muniak, Barbara Dolinska, Rafał Węgrzyn, Kamil Izydorczak

**Affiliations:** ^1^ Faculty of Psychology in Wroclaw SWPS University of Social Sciences and Humanities Wroclaw Poland; ^2^ Warsaw Faculty, Centre for Research on Social Relations SWPS University of Social Sciences and Humanities Warsaw Poland; ^3^ Faculty of Social Sciences University of Opole Opole Poland

**Keywords:** applied social psychology, COVID‐19, health and well‐being, media intervention program, unrealistic optimism bias

## Abstract

Unrealistic optimism is the tendency to perceive oneself as safer than others in situations that equally threaten everybody. By reducing fear, this bias boosts one's well‐being; however, it is also a deterrent to one's health. Three experiments were run in a mixed‐design on 1831 participants to eliminate unrealistic optimism (measured by two items—probability of COVID‐19 infection for oneself and for others; within‐subjects) toward the probability of COVID‐19 infection via articles/videos. A between‐subject factor was created by manipulation. Ostensibly, daily newspaper articles describing other people diligently following medical recommendations (experiment 1) and videos showing people who did not follow these recommendations (experiment 2) reduced unrealistic optimism. The third experiment, which included both articles and videos, replicated these results. These results can be applied to strategies for written and video communications that can be used by governments and public health agencies as best practices concerning not only COVID‐19 but also any subsequent public health threat while promoting proactive, optimal, and healthy functioning of the individual.

## INTRODUCTION

Throughout human history, the world has been viewed as unpredictable. From an evolutionary perspective, early humans favored positive biases in order to deal with an environment that was difficult to predict as optimism provides greater benefits and fewer costs than pessimism (Haselton & Nettle, 2006; Jefferson et al., 2017). This evolutionary preference continues to exist in the case of social comparisons, privileging the person making them, framing the future in a more flattering way. This distortion is defined by Weinstein—creator of this concept—as “unrealistic optimism” where “negative events are less likely to happen to them than to others, and positive events are more likely to happen to them than to others” (Weinstein, 1980, p. 807).

Importantly, a careful reader might raise a question about the general distinction of optimism and unrealistic optimism. It seems that the most important borderline point is that research shows that people may be unrealistically optimistic about certain potential situations in the future and not optimistic (or even pessimistic) about others (e.g., Hilton et al., 2011; Shepperd et al., 1996). It suggests therefore that unrealistic optimism should not be treated as a personality trait.

Another difference is that, while optimism generally means a positive outlook on one's personal life (e.g., Carver et al., 2010; Gallagher et al., 2013), unrealistic optimism has a solely comparative character. For example, on the one hand, a student holding an unrealistic optimism bias might expect serious heart failure before becoming 40 years old; on the other hand, this person may expect other colleagues from the university to be even more exposed to this danger making it more probable for them.

Of course, from the point of view of a specific person, it is possible that a specific student might live an extraordinarily healthy life (exercise and diet), often undergoing medical screenings, making her/him less exposed to heart failure in comparison to other students. This perception becomes unrealistic if most of her/his colleagues hold the same perception in which every single person would expect lower chances of heart failure than others in the class. Thus, for this reason, the adjective “unrealistic” used in Weinstein's definition does not refer to objectively grounded reasons making someone feel less threatened by future negative events, but to unrealistic comparisons in which many individuals processing such comparisons are delusional about such future threatening events.

## UNREALISTIC OPTIMISM AND ITS POSITIVE IMPACT ON WELL‐BEING

Over four decades, studies have demonstrated that unrealistic optimism is a robust and widespread phenomenon. People believe that they are immune to adversity, such as road accidents (Rutter et al., 1998), divorce (Lin & Raghubir, 2005), or substance abuse (Nezlek & Zebrowski, 2001), while others are not. At the same time the same people also believe that good things and events such as passing exams (Lewine & Sommers, 2016) or earning a high salary after graduation (Shepperd et al., 1996) are more likely to happen to them than to their peers. From this perspective, it is clear that unrealistic optimism has an important role in protecting well‐being by reducing fear and anxiety (e.g., Hoorens, 1995), preserving a sense of control (e.g., Taylor, 1989), and helping confront potential threats (e.g., Shepperd et al., 1996).

## UNREALISTIC OPTIMISM AND ITS NEGATIVE IMPACT ON HEALTH

At the same time, unrealistic optimism leads to negative consequences for one's health. For example, compared to cigarette smokers who made accurate personal risk estimations, unrealistically optimistic smokers, who underestimated the likelihood of getting cancer, were less likely to quit smoking, and more likely to perceive cigarettes as less harmful than they truly are (Dillard et al., 2006). Weinstein et al. (2005) found that unrealistically optimistic smokers underestimate the chances of developing lung cancer in comparison to other smokers and non‐smokers, leading to a greater risk of suffering from this disease. In a longitudinal study, college students who were unrealistically optimistic about alcohol problems were more likely to experience alcohol‐related problems at 6‐month, 12‐month, and 18‐month follow‐ups (Dillard et al., 2009). Similarly, unrealistically optimistic women were less likely to undergo mammographic screening (McCaul et al., 1996), leading to a greater risk of breast cancer. Hanoch et al. (2019) demonstrated that patients overestimate their likelihood of benefiting from treatment and underestimate their likelihood of risk (such as side effects), resulting in unnecessary, sometimes counterproductive, or even harmful, and costly medical interventions.

In conclusion, unrealistic optimism is at the same time beneficial for protecting well‐being (it helps to rationalize and manage fears) but at the same time could be harmful for one's health. For example, Texans, on the one hand, reported a lower likelihood of becoming infected by COVID‐19 and felt less stressed, depressed and helpless. On the other hand, they behaved irresponsibly in terms of their health, for example, exposed themselves to the COVID‐19 infection during long travels and many meetings stemming from them (Gassen et al., 2021). From a health perspective, it is crucial to create and test intervention programs that address at least the reduction, if not elimination, of this dangerous situational bias. It is precisely this that is the aim of the present study.

## IS IT POSSIBLE TO REDUCE OPTIMISM BIAS TO PROTECT HEALTH?

In Weinstein's study, participants listed factors that influenced the probability of something happening to them (1983). Subsequently, some of the participants were given copies of lists generated by others. In this second group, the level of unrealistic optimism diminished, leading the author to stipulate that egocentrism may be a key factor in fueling unrealistic optimism. A more recent study showed that egocentrism was reduced by providing narratives—short autobiographies of people affected by a health problem (Kim & Niederdeppe, 2016). It is clear that, on the one hand, people focus on factors that increase their chances of avoiding harm and give themselves credit for favorable factors but fail to give similar credit to others; on the other hand, they do not consider circumstances that may favor other people. Considering these two aspects, reducing egocentrism is the first starting point for creating models of media interventions targeted at the reduction of unrealistic optimism. From this perspective, it would be reasonable to expect that delivering news about the severity of an illness affecting others would reduce egocentrism and would thus reduce unrealistic optimism.

Unrealistic optimism has been shown to be closely related to controllability (e.g., Weinstein, 1980). People who demonstrate unrealistic optimism may believe that they themselves exhibit more behavior aimed at avoiding harm (e.g., infection) than other people. On this basis, we expected that delivering media information about others' active actions oriented toward protecting their health would reduce or even eliminate participants' unrealistic optimism.

## TESTING THE INTERVENTION—THE GOAL OF THE STUDY

The present study, to the best of our knowledge, is the first to systematically and experimentally test different mass‐media communications aimed at the situational (not to influence optimism as a trait) reduction of unrealistic optimism bias. In this study, we tested both written and video channels for their effect on situational unrealistic optimism reduction. The basis for testing our expectations was the ongoing COVID‐19 pandemic. In previous studies it was already shown that citizens of many different countries report unrealistic optimism while accessing their own and others' chances for the infection (Dolinski et al., 2020; Druică et al., 2020; Kulesza et al., 2020). In our studies we planned to test the reduction in situational unrealistic optimism. Since unrealistic optimism about COVID‐19 is based on the person's misconception that other people exhibit less health‐promoting behavior than he or she does, we expected that delivering media information about others who strictly adhere to following recommendations and restrictions should eliminate one's feelings of unique invulnerability (Perloff & Fetzer, 1986). We expected to reach that goal by reducing egocentrism (Weinstein, 1983), and reminding participants of others' active actions (Ross & Sicoly, 1979).

The aforementioned expected pattern of results led us to pose a general hypothesis: information provided to respondents that others actively involve themselves in various health‐protection behaviors oriented against COVID‐19 will reduce their level of unrealistic optimism regardless of the source (written/article vs. video).

In Experiment 1, participants read newspaper articles about other people complying with (or defying) medical recommendations. In Experiment 2, participants watched videos depicting others' behaviors as they followed (or ignored) these recommendations. Since—unexpectedly—the results from these two experiments were contradictory, Experiment 3 was designed to explore discrepancies.

## GENERAL METHODS

### Sample

The sample size was determined by feasibility criteria. We strove to recruit as many participants as possible, given the limited time and size of our sampling pool. Participants of all three experiments were subscribers of the newsletter of the local university in Poland. The subscribers' sample consisted of people who participated in various events for the general public and followers of the university's social media. All participants were Polish‐speaking and presumably current Polish residents. Participants were recruited through email invitations containing a link to the online experiment with embedded randomization. The invitation was sent only once to each participant; thus, they could not be re‐invited to another experiment. Additionally, it was impossible to participate in the online experiment more than once from the same IP address.

While planning experiments, we paid close attention to rule out two variables that could affect our reasoning stemming from the gathered data. First, all participants were questioned about their COVID‐19 infection status (positive test). This precaution was absolutely crucial for the planned experiments since the theoretical pillar was unrealistic optimism, and prior COVID‐19 infection would make participants realistically assess that the probability of future infection would be lower for themselves and greater for others. In these cases, such a disproportion would be assessed; however, one cannot speak of unrealistic optimism but of realistic COVID‐19 infection risk assessment grounded in data. To control this variable, data from all participants who had positive COVID‐19 results were not taken into consideration. Second, experiments were conducted during the period in which vaccines were not accessible for participants. It is clear that being vaccinated would make participants expect lower chances for future COVID‐19 infection compared to those who were not vaccinated and such expectations would have their roots not in unrealistic optimism bias, but in realistically evaluating vaccine efficacy.

### Measures

Unrealistic optimism bias was assessed by two questions: (Q1) *What is the probability that you will be infected with the novel coronavirus (SARS‐CoV‐2/COVID‐19)?* (Q2) *What is the probability that a friend of yours (of your age and gender) will become infected with the novel coronavirus (SARS‐CoV‐2/COVID‐19)?* The respondents rated their answers on an 11‐point scale (1 = *Absolutely impossible*; 11 = *Quite certain*). At the end of each experimental procedure, the participants were asked to indicate their age and gender.

### Statistical analysis and open practices

JASP statistical software (Version 0.13.1) was used to conduct the analysis with a combination of the R programming language implemented in RStudio v. 3.6.1 (R Core Team, 2019) with a “tidyverse” package (Wickham et al., 2019) for the creation of plots. Post hoc power test in G*Power 3.1 (Faul et al., 2009) was run for each analysis. Protocols from the performed power analyses, along with other materials, databases and reports are accessible at the Open Science Framework (OSF; https://osf.io/bsudg).

All experiments were approved by the local ethics committee. Informed consent was obtained from all participants before enrollment in the experimental procedures and data collection.

## EXPERIMENT 1

### Method

#### Participants

In all, 360 participants aged 18–72 (306 women, 51 men, 3 non‐binary: *M*_age_ = 38.89, *SD*_age_ = 9.66) responded to the invitation and 10 participants (7 women, 3 men: *M*_age_ = 28.9, *SD*_age_ = 8.08, ages 22 to 45) were excluded due to at least one missing answer or a declaration of a positive COVID‐19 test result. The final sample consisted of 350 participants (299 women, 48 men, and 3 non‐binary), aged 18–72 years (*M*_age_ = 39.18, *SD*_age_ = 9.56). The participants did not receive any payment or course credit for participating.

#### Procedure

The participants were randomly assigned to the three experimental conditions. In the first two conditions, participants read newspaper articles that were either positive or negative. In the “negative” article condition, 120 participants read an article stating that people were ignoring recommendations or health regulations aimed at preventing the spread of COVID‐19. In the “positive” article condition, 111 participants read an article describing citizens following the anti‐COVID recommendations and regulations dutifully. In the third (control) condition, 119 participants did not read any articles. The articles in the first two conditions were parallel in terms of layout and length, and they were meant to mimic the writing and layout style of a printed local press article. In the next step, participants responded to the two statements assessing optimism bias. Data were collected between September 6–16, 2020.

### Results

Since recent studies by Dolinski et al. (2020) have shown that unrealistic optimism assessment may depend on gender, we ran such comparisons in the first instance.^1^

A 3 × 2 mixed‐design ANOVA was run with one between‐subject factor—experimental condition (3: negative article, positive article, no article, i.e., control group)—and one within‐subject factor—unrealistic optimism bias (2: COVID‐infection risk assessment for “Me” and “Peer”) in order to validate the hypothesis, which states that written sources stating that others actively engage in various health‐related COVID‐19 behaviors reduces the level of unrealistic optimism among participants.

An ANOVA revealed a significant main effect of unrealistic optimism bias, *F*(1, 347) = 45.84, *p* < .001, *η*_p_^2^ = .12 (post hoc test power: 1 − *β* = 1.). Post hoc analysis with a Bonferroni correction revealed that participants presented unrealistic optimism bias as they perceived themselves (*M* = 5.69, *SD* = 2.19) at a lower risk of contracting the COVID‐19 infection than others (*M* = 6.17, *SD* = 2.28; *t* = −6.77, *p*_*bonf*_ < .001; Cohen's *d* = −.36). The main effect of the experimental condition was not significant, *F*(2, 347) = .28, *p* = .754, *η*_p_^2^ = .01 (post hoc test power: 1 − *β* = .99).

**FIGURE 1:**
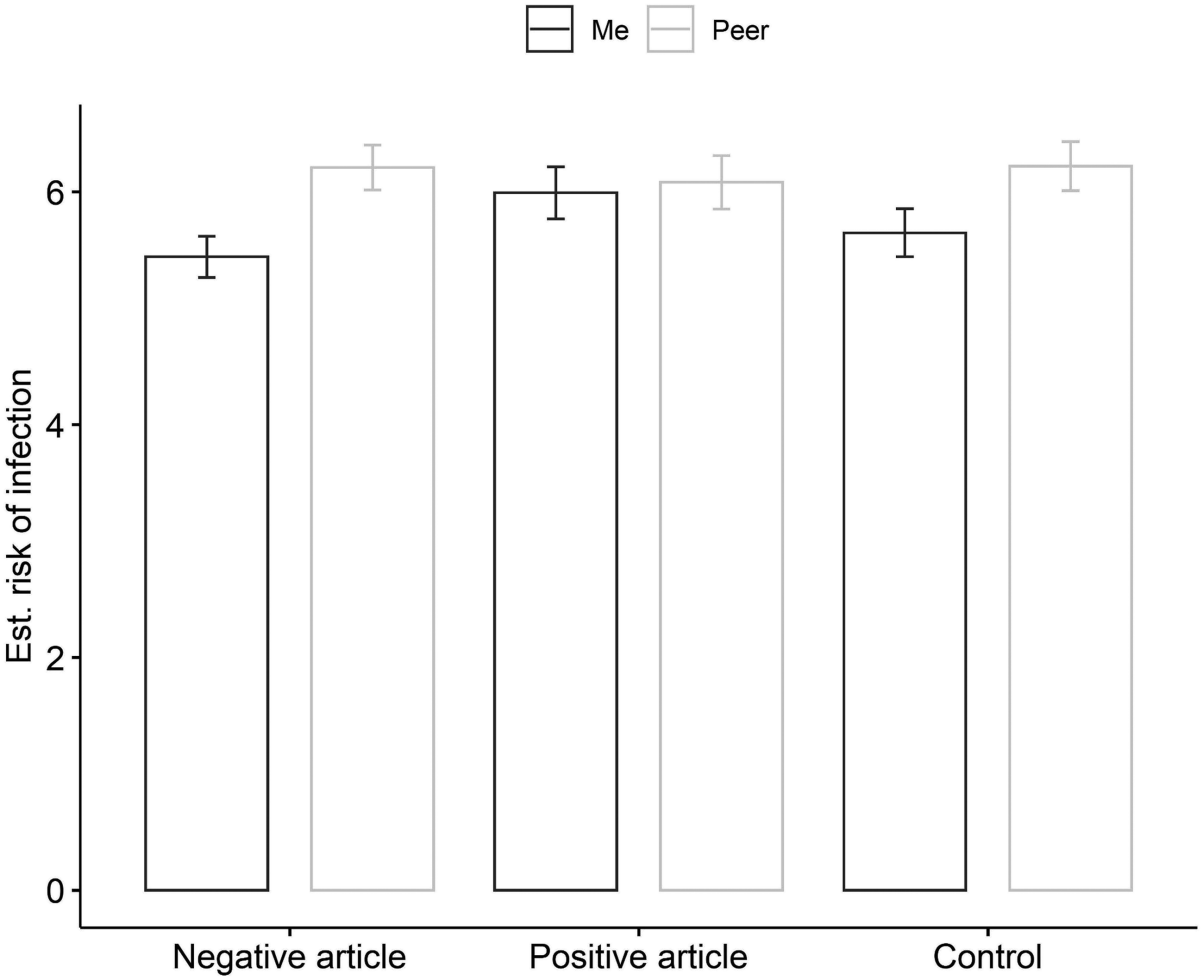
Unrealistic optimism effect in three experimental conditions from study 1. Note: Bars represent mean values, error bars represent standard error of mean

The interaction effect of the experimental condition and optimism bias was significant, *F* (2, 347) = 8.02, *p* < .001, *η*_p_^2^ = .04 (post hoc test power: 1 − *β* = 1.); thus, we performed a post hoc analysis with a Bonferroni correction for all 15 comparisons. We interpreted and reported only three comparisons, based on theoretical expectations. This analysis revealed that unrealistic optimism bias was found in the control condition (with no article). Participants perceived themselves (*M* = 5.65, *SD* = 2.25) at a lower risk of contracting the COVID‐19 than others (*M* = 6.22, *SD* = 2.30; *t* = −4.75, *p*_*bonf*_ < .001; Cohen's *d* = −.25). Unrealistic optimism bias was also found in the negative article condition. Participants perceived themselves (*M* = 5.44, *SD* = 1.94) at a lower risk of contracting the COVID‐19 infection than others (*M* = 6.21, *SD* = 2.13; *t* = −6.39, *p*_*bonf*_ < .001; Cohen's *d* = −.34). In the positive article condition, this bias was not present. Participants perceived themselves (*M* = 5.99, *SD* = 2.36) at the same level of risk of COVID‐19 infection as others (*M* = 6.08, *SD* = 2.42; *t* = −.72, *p*_*bonf*_ = .999; Cohen's *d* = −.04). For more details please see Figure 1 and in supporting information S2.

## DISCUSSION

Previous studies (Dolinski et al., 2020; Druică et al., 2020; Kulesza et al., 2020) have demonstrated that people are unrealistically optimistic about their likelihood of contracting the coronavirus because they perceive themselves as less vulnerable to COVID‐19 than others, and the data gathered in this experiment replicated these findings; however, our experiment showed that this bias disappeared when participants received information about other people following medical recommendations. This result is interesting not only due to the expected predictions but especially for governments regarding the administration of media intervention programs which may be used to change the written narrative to protect citizens' health. In the second experiment, we checked whether the same effect would be observed if the participants received such information in the form of a video (as a proxy for television news and public TV service announcements).

## EXPERIMENT 2

### Method

#### Participants

In all, 600 participants aged 18–73 years (465 women, 131 men, 4 non‐binary: *M*_age_ = 35.01, *SD*_age_ = 11.62) took part in the online experiment, with 32 participants (23 women, 9 men: *M*_age_ = 36.66, *SD*_age_ = 12.45), aged 18–66 years, being excluded from the analysis due to missing answers (a proxy for lack of attention) or a declaration of a positive COVID‐19 test result. The final sample consisted of 568 participants (442 women, 122 men, and 4 non‐binary: *M*_age_ = 34.92, *SD*_age_ = 11.58), 18 to 73 years of age. Participants did not receive any payment or course credit for taking part in the experiment.

#### Procedure

In the second experiment, we aimed to replicate experiment 1 using media in the form of video footage and address one caveat from experiment 1: we planned to extend the previous experiment and also explore if people's optimism bias was affected by the strength of the message—whether it was restricted to extreme (people following or not following safety protocols) and clear messages, or if it also applied to more nuanced communication. To improve the experiment's internal validity, we doubled the number of negative and positive conditions by creating two slightly different videos depicting people ignoring or respecting the precautions against COVID‐19 transmission, respectively.

The “negative” videos depicted citizens who were not following COVID‐preventing regulations and recommendations. In the first “negative” video condition (negative extreme, *n* = 114), the people entered a café without face masks, walked by the disinfectant dispenser without using it, and video showed a group shot of five people crowding in front of the café's counter without respecting the social distance recommendation. None of the people in the scenes wore masks at any point (see materials at OSF). The second negative video condition was aimed at checking the generalizability of the previously reported effect (extreme vs. nuanced; negative nuanced, *n* = 120). Here, instead of all people being without a face mask, one person wore it at least partially: it covered their mouth but not their nose. In the first “positive” video condition (positive extreme, *n* = 108), people entered the café wearing masks properly, stopped to disinfect their hands, and followed social distancing rules by approaching the counter one at a time. However, observing people following recommendations was not easy to notice and required the participants to pay attention (e.g., the hand sanitizer was not visible at first glance). In the second “positive” video condition (positive nuanced, *n* = 106), people in the hand disinfection sequence were recorded with a close‐up on their hands to emphasize that they were following recommendations diligently. In the fifth and last (control) condition, the participants did not watch any video. The participants were randomly assigned to one of five experimental conditions. After the presentation of the video materials (no presentation in the control condition), the participants completed a short questionnaire on unrealistic optimism with identical items to those in the first experiment. The experiment was conducted from September 25, 2020 to October 5, 2020.

### Results

The negative condition extreme video and the negative condition nuanced video did not differ from each other. A mixed‐design ANOVA (2 × 2) with one between‐subject factor—experimental condition (2: negative extreme video and negative nuanced video)—and one within‐subject factor—unrealistic optimism bias (2: COVID‐infection risk assessment for “Me” and “Peer”) revealed an insignificant main effect of unrealistic optimism bias, *F* (1, 232) = 0.53, *p* = .470, *η*_p_^2^ > .0. The main effect of the condition was found to be insignificant, *F* (1, 232) = 0.14, *p* = .707, *η*_p_^2^ > .0 as well as the interaction effect of the experimental conditions and optimism bias, *F* (1, 232) = 1.06, *p* = .303, *η*_p_^2^ > .0.

A similar analysis was performed for positive extreme video and positive nuanced video. An ANOVA (2 × 2) revealed a significant main effect of unrealistic optimism bias, *F* (1, 212) = 65.05, *p* < .001, *η*_p_^2^ = .24. Post hoc analysis with a Bonferroni correction revealed that participants rated their chances of contracting COVID‐19 (*M* = 5.18, *SD* = 2.19) significantly lower compared to the likelihood of others getting infected (*M* = 5.91, *SD* = 2.47; *t*  −8.07, *p*_bonf_ < .001; Cohen's *d*  −.55). The main effect of the experimental condition was found to be insignificant, *F* (1, 212) = 0.71, *p* = .401, *η*_p_^2^ > .0 as well as the interaction effect of the experimental conditions and optimism bias, *F* (1, 212) = 0.11, *p* = .740, *η*_p_^2^ > .0.

Given the lack of significant main effects of conditions, we combined positive extreme video and positive nuanced video conditions into one positive video condition (*n* = 214) and negative extreme video and negative nuanced video conditions into one negative video condition (*n* = 234). We analyzed data separately for all five experimental conditions (5 × 2 mixed design ANOVA), which may be found in supporting information S3.

Thus, in order to validate the hypothesis, a 3 × 2 mixed‐design ANOVA was conducted with one between‐subject factor—a video (3: negative condition, positive condition, control group)— and one within‐subject factor— unrealistic optimism bias (2: COVID‐infection risk assessment for “Me” and “Peer”). An ANOVA revealed a significant main effect of unrealistic optimism bias, *F*(1, 565) = 66.42, *p* < .001, *η*_p_^2^ = .11 (post hoc test power: 1 − *β* = 1.). Post hoc analysis with a Bonferroni correction revealed that participants perceived themselves (*M* = 5.82, *SD* = 2.38) at a lower risk of contracting COVID‐19 than others (*M* = 6.27, *SD* = 2.44; *t*  −8.15, *p*_*bonf*_ < .001; Cohen's *d* = −.34).

The main effect of the experimental condition was significant, *F*(2, 565) = 11.92, *p* < .001, *η*_p_^2^ = .04 (post hoc test power: 1 − *β* = 1.). Thus, we performed a post hoc analysis with a Bonferroni correction for all three comparisons. Comparisons between the groups showed that the average COVID‐infection risk assessment differed significantly between the positive video (*M* = 5.54, *SE* = .15) and negative video (*M* = 6.13, *SE* = .15; *t* = −2.75, *p*_*bonf*_ = .019; Cohen's *d* = −.12). This was also the case for the positive video and control condition (*M* = 6.79, *SE* = .21; *t* = −4.84, *p*_*bonf*_ < .001; Cohen's *d* = −.20), and finally the negative video and control condition (*t* = −2.59, *p*_*bonf*_ = .029; Cohen's *d* = −.11).

**FIGURE 2:**
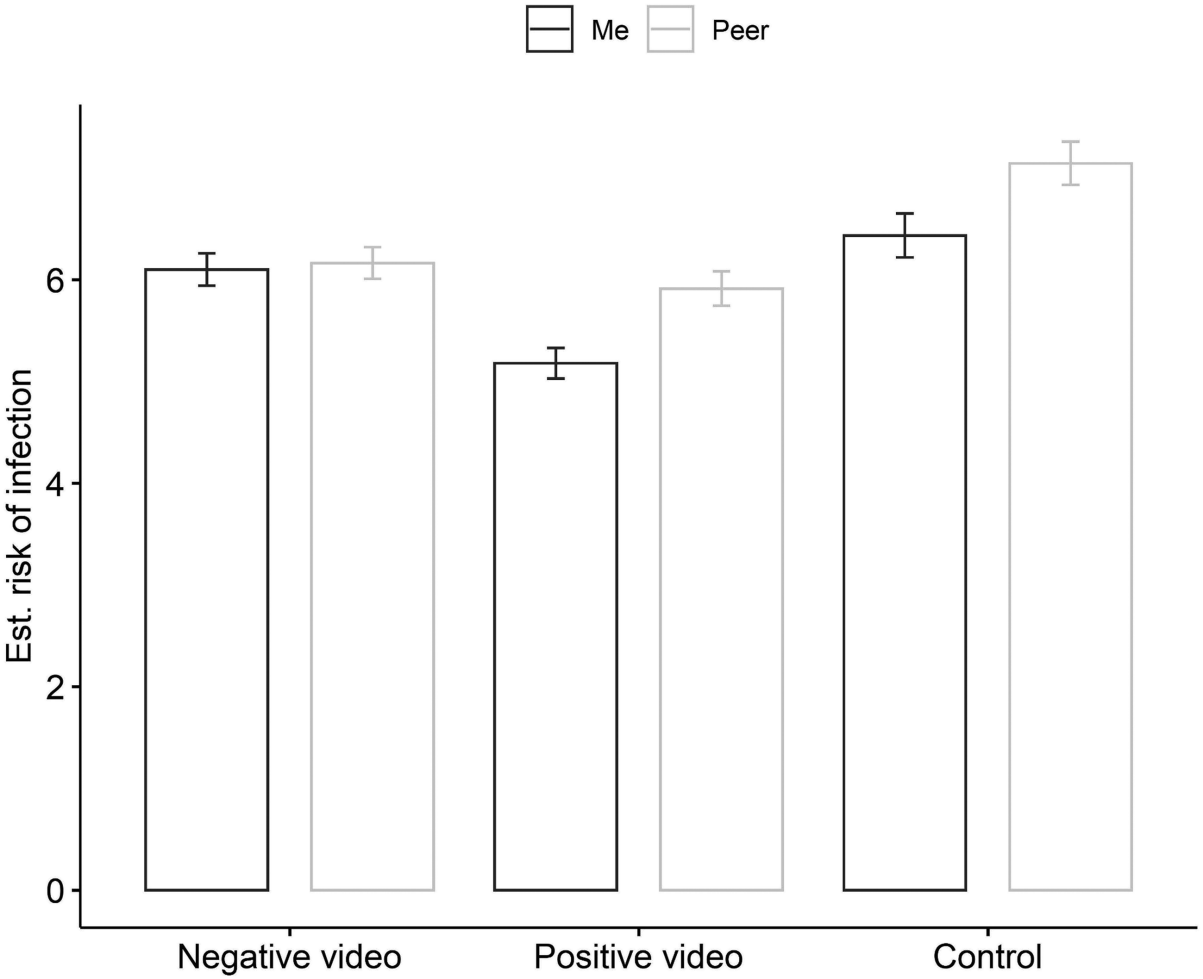
Unrealistic optimism effect in positive and negative movie conditions from study 2. Note: Bars represent mean values, error bars represent standard error of mean

The interaction effect of the experimental condition and optimism bias was significant, *F* (2, 565) = 15.19, *p* < .001, *η*_p_^2^ = .05 (post hoc test power: 1 − *β* = 1.). Thus, a post hoc analysis with a Bonferroni correction for all 15 comparisons was performed. We interpreted and reported only three comparisons based on theoretical expectations. The unrealistic optimism bias was found in the control condition (with no video). Participants perceived themselves (*M* = 6.43, *SD* = 2.38) at a lower risk of contracting COVID‐19 than others (*M* = 7.14, *SD* = 2.32; *t* = −5.52, *p*_*bonf*_ < .001; Cohen's *d* = −.23). Unrealistic optimism bias was also found in the positive video condition. Participants perceived themselves (*M* = 5.18, *SD* = 2.19) at a lower risk of contracting COVID‐19 than others (*M* = 5.91, *SD* = 2.45; *t* = −7.63, *p*_*bonf*_ < .001; Cohen's *d* = −.31). In the negative video condition participants perceived themselves (*M* = 6.09, *SD* = 2.43) at the same level of risk of contracting COVID‐19 as others (*M* = 6.16, *SD* = 2.37; *t* = −.67, *p*_*bonf*_ = .999; Cohen's *d* = −.03). For more details see Figure 2 or supporting information S4.

## DISCUSSION

In Experiment 1, providing participants with information that other people are conscientiously following medical recommendations diminished optimism bias. In Experiment 2, this did not reduce their optimism bias. In fact, in the second experiment, unrealistic optimism was reduced when participants were informed that others behaved irresponsibly, contrary to the results of the first experiment.

There are at least two explanations for this difference. The first explanation refers to the different forms of media used in the experiments (newspaper articles in Experiment 1 and videos in Experiment 2). Based on social cognition, it is assumed that people use two fundamental information‐processing modes to reach a variety of judgments (Chaiken, 1980). Systematic processing is characterized by careful consideration of issues and effortful elaboration of information. Heuristic processing is much less labor intensive and results in the use of learned knowledge structures in the form of simple decision rules or cognitive heuristics (Chaiken & Maheswaran, 1994; Ledgerwood & Callahan, 2012).

Print media (as those we used in Experiment 1) are often treated as more difficult to process/perceive and TV presentation as “easier”, resulting in different levels of effort or elaboration used in processing and encoding the message (Hollander, 2014). For example, it was demonstrated that written presentation of relatively complex material generally resulted in greater comprehension than video presentation (Chaiken & Eagly, 1976). Participants who were shown the text message assessed the information as harder to understand than those who were shown the video message. The participants who were shown the text message also remembered more information than the participants who were shown the video, regardless of the method of memory assessment (recall or recognition). These results are also in line with the results of research showing the relationship between one's knowledge of politics and the media from which they obtain information. It has been demonstrated that text‐based media allows information regarding political events to be retained with a greater degree of accuracy than audiovisual media (Dalrymple & Scheufele, 2007; Eveland et al., 2002).

When it comes to mass media, one should also pay attention to another important distinction between the circumstances in which textual and visual information are processed. As for traditional video messages (used in our Experiment 2), the individual does not influence the way in which information is relayed to them – that is, they do not stop the playback or rewind the video. In these circumstances, the individual's ability to elaborate on information is very limited. However, when it comes to text‐based information, the individual decides the pace at which they receive and process information (reading more slowly or quickly). Furthermore, they can stop, process, and think more deeply about a certain issue at any point and for any length of time before resuming their reading. They can also reread a paragraph in order to rethink a certain issue. Therefore, the reader has the opportunity to elaborate more.

On that basis, one may assume that the article presenting the behavior of others requires effortful analytical processing, so the recipient of the message concluded that there was no reason to believe that they were less likely to contract the infection than other people. In this case, not only would egocentrism be reduced, but the participant would also be motivated to pay attention to the behaviors of others, reducing the effect of one remembering their actions (in this case, preventive action against COVID‐19).

However, watching a video may not cause participants to carefully process information; thus, expected comparisons between “me” and “others” would not be elicited and egocentrism would still be in play. As a result, the article about some people heeding medical recommendations did not make participants think that there was no reason to be optimistic (Experiment 1). Seeing people behaving irresponsibly presumably attracts and holds people's attention because it is concrete and imagery provoking (Nisbett & Ross, 1980). As a result, such information may have led them to conclude that they were in danger (Experiment 2).

An alternative explanation reflects subtle differences in information. The newspaper articles presented to the participants in Experiment 1 read “Citizens follow the rules” versus “Citizens do not follow the rules.” The videos used in Experiment 2 presented the behavior of a small group. It is possible that the information about responsible behavior from the general population led participants to think, “I am no better than others at medical compliance,” which might have reduced unrealistic optimism; giving the participants information about the behavior of a group might not have induced such thinking. Instead, when the participants were informed that a small group of people ignored medical recommendations, and they may have thought that “such irresponsible people in public places can infect me with COVID‐19.”

To check whether either of these two assumptions was an accurate explanation of the discrepancy in the results of Experiment 1 and 2, we conducted a third experiment in which the participants read newspaper articles or watched videos about the population or a group of people following or ignoring medical recommendations. If our first interpretation is correct, the reduction or elimination of unrealistic optimism should occur when the video depicts irresponsible behavior and when the article presents information that people follow medical recommendations. This effect should appear regardless of whether the participants are reading a newspaper article or watching a video depicting the behavior of a small group or general population. However, if our second interpretation is correct, the reduction or elimination of unrealistic optimism should be present only in conditions in which the participants find out that the general population complies with medical recommendations. This effect should appear regardless of whether the participants read a newspaper article or watch a video.

## EXPERIMENT 3

### Method

#### Participants

In all, 1000 participants (806 women, 189 men, 5 non‐binary persons: *M*_age_ = 27.53, *SD*_age_ = 9.96), aged 18–64 years took part in the experiment, and 87 participants (72 women, 14 men, and 1 non‐binary person: *M*_age_ = 27.7, *SD*_age_ = 9.0), aged 18 to 54, were excluded due to missing answers (a proxy for lack of attention) or a declaration of a positive COVID‐19 test result. The final sample consisted of 913 participants (734 women, 175 men, and 4 non‐binary: *M*_age_ = 27.51, *SD*_age_ = 10.01), ranging from 18 to 64 years of age. Participants did not receive any payment or course credit to participate in the experiment.

#### Procedure

An online experiment was administered via Qualtrics with negative and positive articles and video materials depicting behaviors of a population or a small group of people. The first two articles were the same as in the first experiment; they described a population as either careful (“positive article‐population,” *n* = 105) or reckless (“negative article‐population,” *n* = 105) when it came to following recommendations about preventing the spread of COVID‐19. In both cases, the population's behaviors were presented to the participants. To address the goals of this experiment, we created a second set of short articles (ostensibly depicting behaviors of small groups of people at weddings) that described careful (“positive article‐small group,” *n* = 105) or reckless (“negative article‐small group,” *n* = 120) behaviors toward preventive rules and regulations. In both cases, the story was about a small group of people in a specific situation. For the first two conditions, the same material as in the second experiment was used— the videos presented a group of people in a café. One depicted people disregarding recommendations while placing an order (“negative video‐small group,” *n* = 109, same video as in negative #1 condition in Experiment 2) and the second presented people following recommendations (“positive video‐small group,” *n* = 96, same video as in positive #2 conditions in Experiment 2). In both videos, the behavior of a small group was presented. We also created a second set of videos, presenting a population in various places following (“positive video‐population,” *n* = 90) or not following the recommendations (“negative video‐population,” *n* = 92). Footage was introduced as a general depiction of the population's behavior toward COVID‐19. Finally, in the last (control) condition, 107 participants did not watch any video or read any articles. Participants were randomly assigned to nine groups (eight experimental and one control). The experiment was conducted between November 2 and 12, 2020.

### Results

First, we conducted an analysis to check whether the experimental conditions taken together influenced unrealistic optimism bias. A 9 (two positive videos, two negative videos, two positive articles, two negative articles and no media) × 2 (unrealistic optimism bias assessment: “Me,” “Peer”) mixed‐design ANOVA revealed a significant main effect of unrealistic optimism bias, *F*(1, 904) = 50.65, *p* < .001, *η*_p_^2^ = .05 (post hoc test power: 1 − *β* = 1.). Post hoc analysis with a Bonferroni correction revealed that participants presented optimism bias as they perceived themselves (*M* = 7.21, *SD* = 2.19) as less at risk of contracting COVID‐19 than others (*M* = 7.58, *SD* = 2.14; *t* = −7.12, *p*_*bonf*_ < .001; Cohen's *d* = −.24). The main effect of the experimental condition was not significant, *F*(8, 904) = 1.62, *p* = .115, *η*_p_^2^ = .01 (post hoc test power: 1 − *β* = .99).

The interaction effect of the experimental condition and unrealistic optimism bias was significant, *F*(8, 904) = 2.54, *p* = .01, *η*_p_^2^ = .02 (post hoc test power: 1 − *β* = 1.). Post hoc analysis with a Bonferroni correction was performed, which included all 153 comparisons. We only interpreted and reported nine theoretically meaningful comparisons. This analysis revealed that optimism bias was found in four experimental conditions: (1) Negative article about population (*p*_*bonf*_ = .023; Cohen's *d* = −.13): participants perceived themselves (*M* = 7.20, *SD* = 1.90) at a lesser at risk of contracting the COVID‐19 infection than others (*M* = 7.78, *SD* = 2.03). (2) Positive video about the population (*p*_*bonf*_ = .020; Cohen's *d* = −.13): Participants perceived themselves (*M* = 7.40, *SD* = 2.04) at a lower risk of contracting the COVID‐19 infection than others (*M* = 8.03, *SD* = 1.93). (3) Positive video about the small group (*p*_*bonf*_ = .012; Cohen's *d* = −.13): Participants perceived themselves (*M* = 7.53, *SD* = 2.23) at a lower risk of contracting the COVID‐19 infection than others (*M* = 8.13, *SD* = 2.06). (4) Control condition (no media, *p*_*bonf*_ = .013; Cohen's *d* = −.13): Participants perceived themselves (*M* = 7.03, *SD* = 2.33) at a lower risk of contracting the COVID‐19 infection than others (*M* = 7.63, *SD* = 2.31). In all other conditions (both positive articles and both negative videos as well as in the negative article about the small group), the presence of this bias was not found (*p*_*bonf*_ = .999). For more details see supporting information S5.

Second, we conducted an analysis to check whether the discrepancy between results from Experiment 1 and Experiment 2 could be ascribed to different media types (article vs. video) or different contexts (small group vs. population). In the first step, we checked whether the media type (article vs. video) was responsible for the aforementioned differences. A 2 (media type: article vs. video) × 2 (behavior toward recommendations: positive‐following rules and recommendations vs. negative‐not complying with) × 2 (unrealistic optimism bias assessment: “Me,” “Peer”) mixed‐design ANOVA was conducted.

**FIGURE 3:**
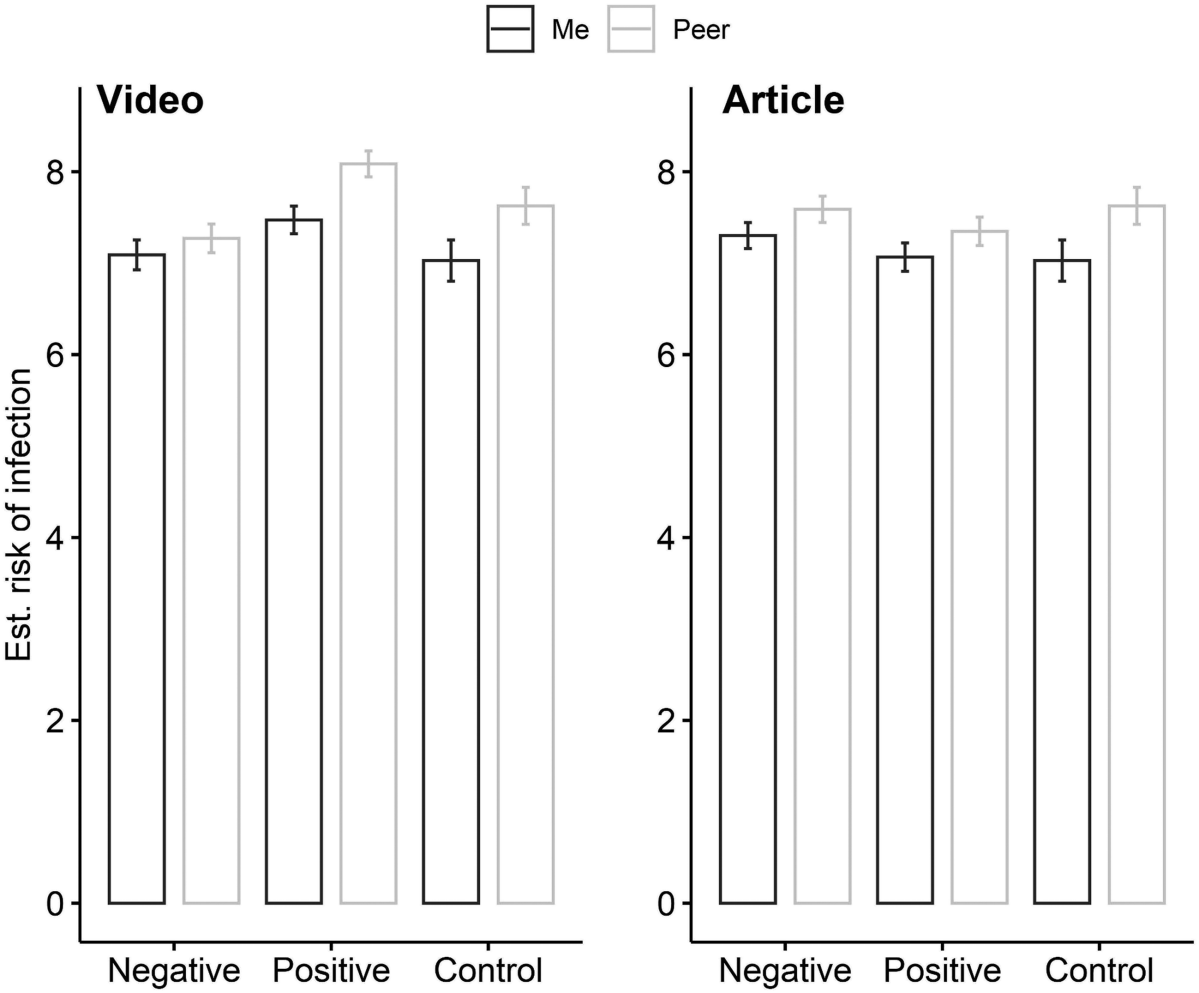
Interaction effect from study 3: Unrealistic optimism bias assessment * behavior toward recommendation (positive, negative) * media type (video, article). Note: Bars represent mean values, error bars represent standard error of mean

The interaction effect of media type, behaviors toward recommendations, and unrealistic optimism bias turned out to be significant, *F*(1, 802) = 4.35, *p* = .037, *η*_p_^2^ > .0 (post hoc test power: 1 − *β* = .99). See main effects and all interaction effects in supporting information S6. In the case of video material, the unrealistic optimism bias was influenced by the type of presented behavior—bias was present when the people in the video followed rules and recommendations and was absent otherwise. In the case of the article, unrealistic optimism bias was not influenced by the type of behavior that the articles described (see Figure 3).

In the second step, we checked whether the context (small group vs. population) influenced our results. A 2 (context: small group vs. population) × 2 (behavior toward recommendations: positive‐following rules and recommendations vs. negative‐neglecting) × 2 (unrealistic optimism bias assessment: “Me,” “Peer”) mixed‐design ANOVA was conducted. The interaction effect of context, behavior toward recommendations, and unrealistic optimism bias was not significant, *F*(1, 802) = 1.33, *p* = .249, *η*_p_^2^ > .0 (post hoc test power: 1 − *β* = .88). See main effects and all interaction effects in supporting information S7. The presence of the unrealistic optimism bias did not depend on any particular combination of context and type of behavior.

### Discussion

The third experiment was conducted to explain the contrary results obtained from Experiment 1 and Experiment 2. The pattern of results obtained in Experiment 3 clearly shows the key role played by the type of media (article vs. video) presented to participants. When participants were presented with newspaper articles about people following medical recommendations, they did not demonstrate unrealistic optimism. This effect was observed regardless of whether the behavior of citizens or friends was described. A similar effect (lack of unrealistic optimism bias) was also observed when participants were confronted with a video depicting people who were *not* following medical recommendations.

The role of the second factor (small group vs. population) differentiating the procedures of Experiment 1 and Experiment 2 turned out to be of very little importance and was only relevant when the newspaper article mentioned people who did not follow medical recommendations. The above‐mentioned pattern of the results obtained in Experiment 3 perfectly matches previous assumptions that reading newspaper articles is more effortful than watching videos.

Only one result obtained by us did not fit this reasoning. Unrealistic optimism was not observed when an article portrayed a small group that did not follow medical recommendations. Most likely, people in such conditions do not treat the incoming information as enabling them to compare themselves with the “average person.” On the contrary, they use direct emotional inferences from observed scenes and think “because of such people I can get infected.” Future studies should address this issue.

## GENERAL DISCUSSION

Research on the role of the mass‐communication in a public health context usually focuses on comparing the content of information delivered through a written channel and through a video channel (Awofeso et al., 2019; Catalan‐Matamoros & Peñafiel‐Saiz, 2019; Patterson et al., 2016) and on the frequency with which the information reaches recipients through these channels (e.g., Gombeski et al., 1982; Hu et al., 1989). Occasionally, however, studies focus on the relation between particular media used in a mass‐communication and the effectiveness of the health campaign. Schooler et al. (1998) examined comparative effects of different health campaign channels used in a health risk reduction project. Printed messages appeared to have a stronger impact on the amount of participants' health knowledge than TV. Cheung et al. (2017), on the contrary, demonstrated the advantage of video channels over a text during a computer‐tailored intervention for obesity prevention. Importantly, however, to the best of our knowledge, there is no research on what specific content is better to present via the video channel, and which with the use of text. Therefore we believe that our research, at least partially, fills this gap.

The results of Experiment 1 demonstrated an elimination of unrealistic optimistic bias when participants read a text about people following restrictions and recommendations for the prevention of the spread of COVID‐19. In Experiment 2, observing a video of people who did not follow the recommendations eliminated participants' unrealistic optimism. Experiment 3 replicated these results, presenting not only the robustness of the effect, but also showing that the key factor was media type (newspaper or video) through which other people's careful or reckless behavior regarding rules and regulations is presented. This may be due to the fact that reading articles requires more effortful information processing than watching a video.

To the best of our knowledge, this strand of research grounded in research and theory provided by Weinstein (1980, 1983) has proven to be effective in media communication during the global health crisis. In our opinion, the results gathered in this research are not limited to the COVID‐19 pandemic period, but to other future public health threats where the behavior of others may affect one's health (i.e., reduction of risk for infection; death), and vice versa (where one's behavior influences others' chances of staying healthy or alive).

### Practical implications

To dismantle unrealistic optimism, it is crucial to at least reduce egocentrism by exposing people to written descriptions of fellow citizens following health recommendations. In the case of video footage, it was necessary to depict people not following recommendations to eliminate unrealistic optimism. Mass communication used by governments, and agencies responsible for public health should adjust messages accordingly.

While carrying out massive interventions targeted toward unrealistic optimism reduction, one should keep in mind that *optimism* for protecting and maintaining one's health is generally beneficial (since the long‐term consequences of optimism on well‐being are ambiguous; de Meza & Dawson, 2021). After coronary bypass surgery, faster recovery has been reported by optimists who, at the same time, experienced less pain (Fitzgerald et al., 1993). Optimists, compared to pessimists, exhibit high blood pressure less often (Räikkönen et al., 1999). In both children (Ey et al., 2005) and adults (Alloy et al., 2009), optimism is linked to lower depression by buffering the effects of life stress, suggesting that it is functional in maintaining one's health (e.g., Taylor & Brown, 1988).

Finally, perhaps the golden rule for making decisions about what to do with optimism in the domain of public and global health is that a small dose of optimism is beneficial, whereas extreme (unrealistic) optimism is harmful (e.g., de Meza & Dawson, 2021). It may be similar to the difference between illusions and delusions—as Baumeister (1989) suggests, one may treat illusions as positive, but without any doubt, delusions negatively impact one's life.

### Limitations

A critical reader of this manuscript might pose a counter hypothesis that perhaps the participants were already vaccinated against COVID‐19, and thus, no unrealistic optimism bias may be present; this would be a justified estimation, as vaccinated people are less exposed to the danger posed by this virus. This limitation was not applicable to our experiment. We planned and ran (fall of 2020) our studies prior to when vaccines were distributed (January 2021).

The basic limitation of our research stems from its experimental nature. The participants knew that they were taking part in the experiment. Additionally, we were unable to control what other pandemic information our participants had come into contact with (for example: if anybody close to the participant died from this virus).

Another limitation of our research is that we did not conduct it on a fully representative sample. The participants were people who used various free online educational forums (webinars, lecture presentations, and discussion forums) organized by the university; thus results and recommendations are applicable for this specific sample.

Our experiments were run in the setting of the pandemic. In our opinion these results may be applied in other areas of the public and global health domain; However, future replications in other health contexts (other viruses, diseases, etc.) should be conducted. With the present data in hand, we cannot rule out the possibility that the results presented above are restricted solely to the COVID‐19 pandemic.

## CONFLICT OF INTEREST

All authors declare a lack of conflict of interest with respect to the research, authorship, and/or publication of this article.

## ETHIC STATEMENT

All experiments were approved by the local ethics committee. Informed consent was obtained from all participants before enrollment in the experimental procedures and data collection.

## Supporting information


**Data S1.** Supporting Information

**Table S1.** *Summary of results from Study 1*

**Table S1.** *Post‐hoc comparisons for the main effect of experimental conditions from Study 2*
**Table S2.** *Summary of results from Study 2*

**Table S1.** *Summary of results from Study 2*

**Table S1.** *Summary of the results from Study 3: Unrealistic optimism bias in all experimental conditions*

**Table S1.** *Within‐subjects effects from Study 3: Unrealistic optimism bias assessment, behavior towards recommendations, and media type*
**Table S2.** *Between‐subjects effects from Study 3: Behavior towards recommendations and media type*

**Table S1.** *Within‐subjects effects from Study 3: Unrealistic optimism bias assessment, behavior towards recommendations, and context*
**Table S2.** *Between‐subjects effects from Study 3: Behavior toward recommendations and context*

## Data Availability

The data set underlying this study with all materials is available from the Open Science Framework database. The URL necessary to access our data is: https://osf.io/bsudg

## ENDNOTE

^1^
In all three experiments there was no main effect of gender (first experiment: *F*(1, 345) = .43, *p* = .512, η_p_^2^ > .0; second: *F*(1, 562) = .16, *p* = .693, η_p_^2^ > .0; third: *F*(1, 907) = 1.32, *p* = .250, η_p_^2^ > .0). Therefore, in each subsequent analyses, gender was not taken into consideration. For more details see Supporting Material 1.
