# Supplementary material for: Media intervention program for reducing unrealistic optimism bias: The link between unrealistic optimism, well‐being, and health
Source: Appl Psychol Health Well Being. 2021 Oct 24;14(2):499–518. doi: 10.1111/aphw.12316 (PMC9298214; doi:10.1111/aphw.12316)

# **Study 1**

A mixed-design ANOVA was run with one between-subject factor — gender (2: male, female) —and one within-subject factor — unrealistic optimism bias (2: COVID-infection risk assessment for "Me" and "Peer"). We decided not to include non-binary participantas in the analysis, due to the extremely small number of participants decapping this gender (*n* = 3).

## **Repeated Measures ANOVA**

| **Within Subjects Effects** | | | | | | | | | | | | | |
| --- | --- | --- | --- | --- | --- | --- | --- | --- | --- | --- | --- | --- | --- |
| **Cases** | | **Sum of Squares** | | **df** | | **Mean Square** | | **F** | | **p** | | **η² _p_** | |
| RM Factor 1 |  | 28.502 |  | 1 |  | 28.502 |  | 31.653 |  | < .001 |  | 0.084 |  |
| RM Factor 1 ✻ sex |  | 1.672 |  | 1 |  | 1.672 |  | 1.857 |  | 0.174 |  | 0.005 |  |
| Residuals |  | 310.659 |  | 345 |  | 0.900 |  |  |  |  |  |  |  |
|  | | | | | | | | | | | | | |
| *Note.*  Type III Sum of Squares | | | | | | | | | | | | | |

| **Between Subjects Effects** | | | | | | | | | | | | | |
| --- | --- | --- | --- | --- | --- | --- | --- | --- | --- | --- | --- | --- | --- |
| **Cases** | | **Sum of Squares** | | **df** | | **Mean Square** | | **F** | | **p** | | **η² _p_** | |
| sex |  | 3.953 |  | 1 |  | 3.953 |  | 0.432 |  | 0.512 |  | 0.001 |  |
| Residuals |  | 3159.444 |  | 345 |  | 9.158 |  |  |  |  |  |  |  |
|  | | | | | | | | | | | | | |
| *Note.*  Type III Sum of Squares | | | | | | | | | | | | | |

###

###

### **Descriptives**

|  | | | | | | | | | |
| --- | --- | --- | --- | --- | --- | --- | --- | --- | --- |
| **RM Factor 1** | | **sex** | | **Mean** | | **SD** | | **N** | |
| Level 1 |  | FEMALE |  | 5.736 |  | 2.140 |  | 299 |  |
|  |  | MALE |  | 5.375 |  | 2.540 |  | 48 |  |
| Level 2 |  | FEMALE |  | 6.181 |  | 2.201 |  | 299 |  |
|  |  | MALE |  | 6.104 |  | 2.762 |  | 48 |  |
|  | | | | | | | | | |

| **Post Hoc Comparisons - RM Factor 1** | | | | | | | | | | | | | |
| --- | --- | --- | --- | --- | --- | --- | --- | --- | --- | --- | --- | --- | --- |
|  | |  | | **Mean Difference** | | **SE** | | **t** | | **Cohen's d** | | **p _bonf_** | |
| Level.1 |  | Level.2 |  | -0.587 |  | 0.104 |  | -5.626 |  | -0.302 |  | < .001 |  |
|  | | | | | | | | | | | | | |
| *Note.*  Cohen's d does not correct for multiple comparisons. | | | | | | | | | | | | | |
| *Note.*  Results are averaged over the levels of: sex | | | | | | | | | | | | | |


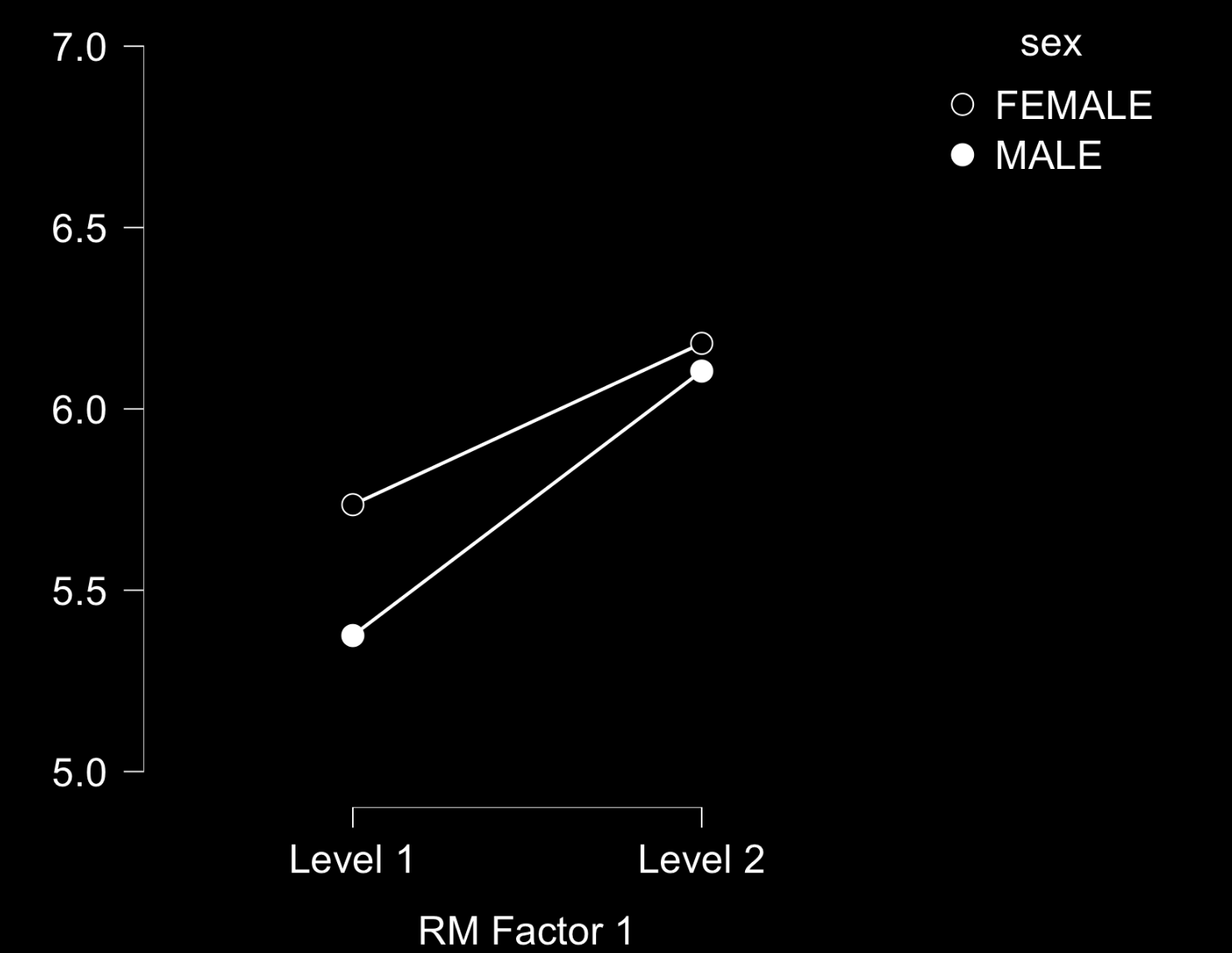


# **Study 2**

A mixed-design ANOVA was run with one between-subject factor — gender (2: male, female) —and one within-subject factor — unrealistic optimism bias (2: COVID-infection risk assessment for "Me" and "Peer"). We decided not to include non-binary participantas in the analysis, due to the extremely small number of participants decapping this gender (*n* = 4).

**Repeated Measures ANOVA**

| **Within Subjects Effects** | | | | | | | | | | | | | |
| --- | --- | --- | --- | --- | --- | --- | --- | --- | --- | --- | --- | --- | --- |
| **Cases** | | **Sum of Squares** | | **df** | | **Mean Square** | | **F** | | **p** | | **η² _p_** | |
| RM Factor 1 |  | 49.490 |  | 1 |  | 49.490 |  | 47.576 |  | < .001 |  | 0.078 |  |
| RM Factor 1 ✻ sex |  | 2.149 |  | 1 |  | 2.149 |  | 2.066 |  | 0.151 |  | 0.004 |  |
| Residuals |  | 584.605 |  | 562 |  | 1.040 |  |  |  |  |  |  |  |
|  | | | | | | | | | | | | | |
| *Note.*  Type III Sum of Squares | | | | | | | | | | | | | |

| **Between Subjects Effects** | | | | | | | | | | | | | |
| --- | --- | --- | --- | --- | --- | --- | --- | --- | --- | --- | --- | --- | --- |
| **Cases** | | **Sum of Squares** | | **df** | | **Mean Square** | | **F** | | **p** | | **η² _p_** | |
| sex |  | 1.650 |  | 1 |  | 1.650 |  | 0.156 |  | 0.693 |  | 2.768e -4 |  |
| Residuals |  | 5958.764 |  | 562 |  | 10.603 |  |  |  |  |  |  |  |
|  | | | | | | | | | | | | | |
| *Note.*  Type III Sum of Squares | | | | | | | | | | | | | |

| **Descriptives** | | | | | | | | | |
| --- | --- | --- | --- | --- | --- | --- | --- | --- | --- |
| **RM Factor 1** | | **sex** | | **Mean** | | **SD** | | **N** | |
| Level 1 |  | FEMALE |  | 5.871 |  | 2.428 |  | 442 |  |
|  |  | MALE |  | 5.672 |  | 2.232 |  | 122 |  |
| Level 2 |  | FEMALE |  | 6.274 |  | 2.452 |  | 442 |  |
|  |  | MALE |  | 6.287 |  | 2.386 |  | 122 |  |
|  | | | | | | | | | |

| **Post Hoc Comparisons - RM Factor 1** | | | | | | | | | | | | | |
| --- | --- | --- | --- | --- | --- | --- | --- | --- | --- | --- | --- | --- | --- |
|  | |  | | **Mean Difference** | | **SE** | | **t** | | **Cohen's d** | | **p _bonf_** | |
| Level.1 |  | Level.2 |  | -0.509 |  | 0.074 |  | -6.898 |  | -0.290 |  | < .001 |  |
|  | | | | | | | | | | | | | |
| *Note.*  Cohen's d does not correct for multiple comparisons. | | | | | | | | | | | | | |
| *Note.*  Results are averaged over the levels of: sex | | | | | | | | | | | | | |

**
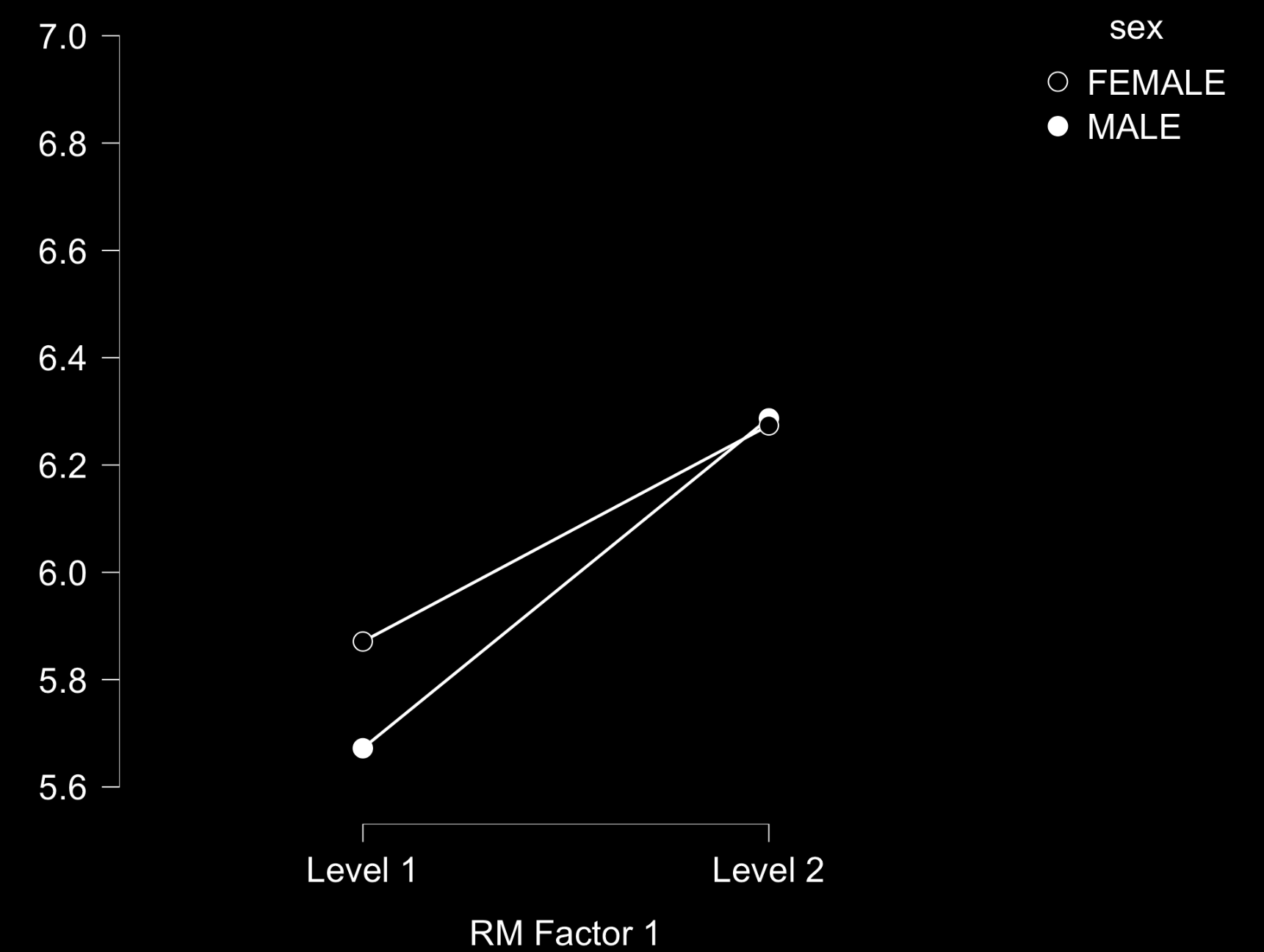
**

# **Study 3**

A mixed-design ANOVA was run with one between-subject factor — gender (2: male, female) —and one within-subject factor — unrealistic optimism bias (2: COVID-infection risk assessment for "Me" and "Peer"). We decided not to include non-binary participantas in the analysis, due to the extremely small number of participants decapping this gender (*n* = 4).

## **Repeated Measures ANOVA**

| **Within Subjects Effects** | | | | | | | | | | | | | |
| --- | --- | --- | --- | --- | --- | --- | --- | --- | --- | --- | --- | --- | --- |
| **Cases** | | **Sum of Squares** | | **df** | | **Mean Square** | | **F** | | **p** | | **η² _p_** | |
| RM Factor 1 |  | 76.756 |  | 1 |  | 76.756 |  | 62.655 |  | < .001 |  | 0.065 |  |
| RM Factor 1 ✻ sex |  | 16.905 |  | 1 |  | 16.905 |  | 13.800 |  | < .001 |  | 0.015 |  |
| Residuals |  | 1111.126 |  | 907 |  | 1.225 |  |  |  |  |  |  |  |
|  | | | | | | | | | | | | | |
| *Note.*  Type III Sum of Squares | | | | | | | | | | | | | |

| **Between Subjects Effects** | | | | | | | | | | | | | |
| --- | --- | --- | --- | --- | --- | --- | --- | --- | --- | --- | --- | --- | --- |
| **Cases** | | **Sum of Squares** | | **df** | | **Mean Square** | | **F** | | **p** | | **η² _p_** | |
| sex |  | 10.778 |  | 1 |  | 10.778 |  | 1.323 |  | 0.250 |  | 0.001 |  |
| Residuals |  | 7390.192 |  | 907 |  | 8.148 |  |  |  |  |  |  |  |
|  | | | | | | | | | | | | | |
| *Note.*  Type III Sum of Squares | | | | | | | | | | | | | |

###

###

| **Descriptives** | | | | | | | | | |
| --- | --- | --- | --- | --- | --- | --- | --- | --- | --- |
| **RM Factor 1** | | **sex** | | **Mean** | | **SD** | | **N** | |
| Level 1 |  | FEMALE |  | 7.297 |  | 2.154 |  | 734 |  |
|  |  | MALE |  | 6.857 |  | 2.326 |  | 175 |  |
| Level 2 |  | FEMALE |  | 7.574 |  | 2.092 |  | 734 |  |
|  |  | MALE |  | 7.623 |  | 2.338 |  | 175 |  |
|  | | | | | | | | | |

| **Post Hoc Comparisons - RM Factor 1** | | | | | | | | | | | | | |
| --- | --- | --- | --- | --- | --- | --- | --- | --- | --- | --- | --- | --- | --- |
|  | |  | | **Mean Difference** | | **SE** | | **t** | | **Cohen's d** | | **p _bonf_** | |
| Level.1 |  | Level.2 |  | -0.521 |  | 0.066 |  | -7.915 |  | -0.263 |  | < .001 |  |
|  | | | | | | | | | | | | | |
| *Note.*  Cohen's d does not correct for multiple comparisons. | | | | | | | | | | | | | |
| *Note.*  Results are averaged over the levels of: sex | | | | | | | | | | | | | |

| **Post Hoc Comparisons - sex ✻ RM Factor 1** | | | | | | | | | | | |
| --- | --- | --- | --- | --- | --- | --- | --- | --- | --- | --- | --- |
|  | |  | | **Mean Difference** | | **SE** | | **t** | | **p _bonf_** | |
| FEMALE, Level.1 |  | MALE, Level.1 |  | 0.440 |  | 0.182 |  | 2.415 |  | 0.095 |  |
|  |  | FEMALE, Level.2 |  | -0.277 |  | 0.058 |  | -4.787 |  | < .001 |  |
|  |  | MALE, Level.2 |  | -0.326 |  | 0.182 |  | -1.789 |  | 0.443 |  |
| MALE, Level.1 |  | FEMALE, Level.2 |  | -0.716 |  | 0.182 |  | -3.934 |  | < .001 |  |
|  |  | MALE, Level.2 |  | -0.766 |  | 0.118 |  | -6.471 |  | < .001 |  |
| FEMALE, Level.2 |  | MALE, Level.2 |  | -0.049 |  | 0.182 |  | -0.271 |  | 1.000 |  |
|  | | | | | | | | | | | |
| *Note.*  P-value adjusted for comparing a family of 6 | | | | | | | | | | | |


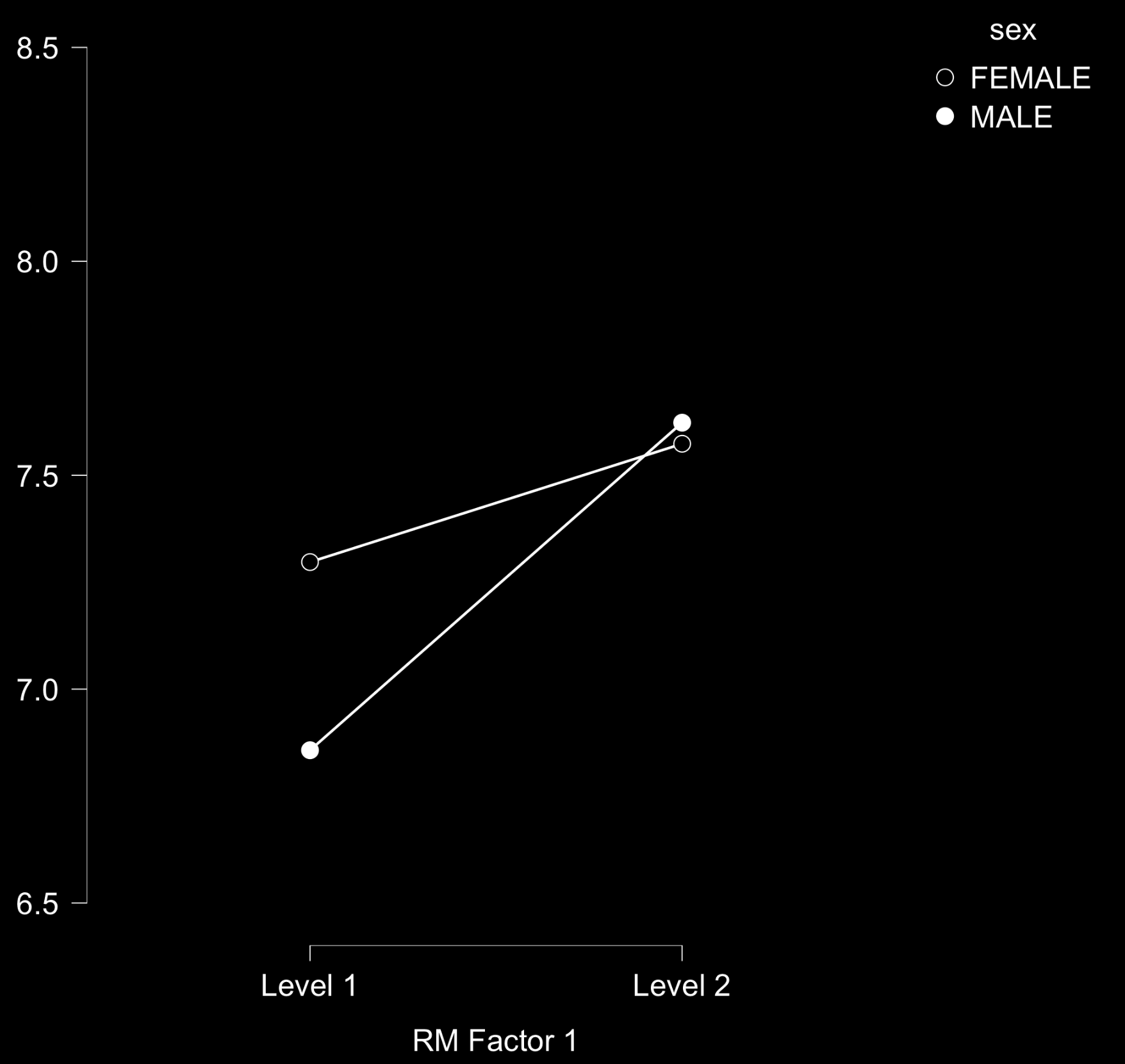

Supplement: Supplementary file 1 — Data S1. Supporting Information [file APHW-14-499-s005.docx]
