# Supplementary material for: Media intervention program for reducing unrealistic optimism bias: The link between unrealistic optimism, well‐being, and health
Source: Appl Psychol Health Well Being. 2021 Oct 24;14(2):499–518. doi: 10.1111/aphw.12316 (PMC9298214; doi:10.1111/aphw.12316)
Supplement: Supplementary file 2 — Table S1. Summary of results from Study 1 [file APHW-14-499-s003.docx]

**Table**

*Summary of results from Study 1*

|  | Unrealistic optimism bias | | | |  | | |
| --- | --- | --- | --- | --- | --- | --- | --- |
|  | Me | | My peer | |  |  |  |
| *Experimental condition* | *M* | *SD* | *M* | *SD* | *t* | *p_bonf_* | Cohen's *d* |
| Control (no article) | 5.65 | 2.25 | 6.22 | 2.30 | -4.75 | <.001 | -.25 |
| Negative article | 5.44 | 1.94 | 6.21 | 2.13 | -6.39 | <.001 | -.34 |
| Positive article | 5.99 | 2.36 | 6.08 | 2.42 | -0.72 | .999 | -.04 |

*Note*. *p*-value adjusted for comparing a family of 15.
