# Supplementary material for: Media intervention program for reducing unrealistic optimism bias: The link between unrealistic optimism, well‐being, and health
Source: Appl Psychol Health Well Being. 2021 Oct 24;14(2):499–518. doi: 10.1111/aphw.12316 (PMC9298214; doi:10.1111/aphw.12316)
Supplement: Supplementary file 3 — Table S1. Post‐hoc comparisons for the main effect of experimental conditions from Study 2 Table S2. Summary of results from Study 2 [file APHW-14-499-s007.docx]

A 5 × 2 mixed-design ANOVA was conducted with one between-subject factor— a video (5: negative video extreme, negative video nuanced, positive video extreme, positive video nuanced, no video i.e., control group)— and one within-subject factor— unrealistic optimism bias (2: COVID-infection risk assessment for “Me” and “Peer”).

ANOVA revealed a significant main effect of unrealistic optimism bias, *F* (1, 563) = 60.54, *p* < .001, η_p_² = .10 (post-hoc test power: 1 - β = 1.). Post-hoc analysis with a Bonferroni correction revealed that participants rated their chances of contracting COVID-19 (*M* = 5.82, *SD* = 2.38) significantly lower compared to of the likelihood of others getting infected (*M* = 6.27, *SD* = 2.44; *t* = −7.78, *p_bonf_* < .001; Cohen’s *d* = −.33).

The main effect of the experimental condition was significant, *F*(4, 563) = 6.16, *p* < .001, η_p_² = .04 (post-hoc test power: 1 - β = 1.). Thus, we performed a post-hoc analysis with a Bonferroni correction for all 10 comparisons. Comparisons between the groups showed that the average COVID-infection risk assessment differed significantly between positive video extreme and the control condition (*t* = -4.57, *p_bonf_* < .001; Cohen’s *d* = −.19), as well as for positive video nuanced and the control condition (*t* = -3.70, *p_bonf_* = .002; Cohen’s *d* = −.16). The differences in COVID-19 infection risk assessment between the other conditions were statistically insignificant. Descriptive statistics are reported in Table 1.

The interaction effect of the experimental conditions and optimism bias was also significant, *F*(4, 563) = 7.84, *p* < .001, η_p_² = .05 (post-hoc test power: 1 - β = 1.). Thus, we performed a post*-*hoc analysis with a Bonferroni correction for all 45 comparisons; however, based on our theoretical expectations, we have interpreted and reported only five theoretically justified comparisons.

This analysis revealed that unrealistic optimism was found in the control condition (no video, *t* = -5.52, *p_bonf_* <.001; Cohen’s *d* = −.23) and in both positive video conditions (positive extreme, *t* = -5.20, *p_bonf_* <.001; Cohen’s *d* = −.22 and positive nuanced, *t* = -5.60, *p_bonf_* <.001; Cohen’s *d* = −.23). For both negative video conditions, the difference in bias was not significant (negative extreme, *t* = .20, *p_bonf_* = .999; Cohen’s *d* = .01 and negative nuanced, *t* = -1.17, *p_bonf_* = .999; Cohen’s *d* = −.05). This result shows that contrary to Study 1, exposing participants to the video depicting people who did *not* follow rules and recommendations reduced unrealistic optimism (see Figure 1). Descriptive statistics are reported in Table 2.

**Table 1**

*Post-hoc comparisons for the main effect of experimental conditions from Study 2*

| *Experimental condition* | | *MD* | *SE* | *t* | *p_bonf_* | Cohen's *d* |
| --- | --- | --- | --- | --- | --- | --- |
| Negative extreme video | Negative nuanced video | 0.11 | 0.30 | 0.38 | .999 | .02 |
|  | Positive extreme video | 0.77 | 0.30 | 2.54 | .112 | .11 |
|  | Positive nuanced video | 0.51 | 0.31 | 1.69 | .927 | .07 |
|  | Control  (no video) | -0.60 | 0.30 | -2.03 | .432 | -.09 |
| Negative extreme video | Positive extreme video | 0.66 | 0.30 | 2.20 | .284 | .09 |
|  | Positive nuanced video | 0.40 | 0.30 | 1.33 | .999 | .06 |
|  | Control  (no video) | -0.71 | 0.29 | -2.44 | .149 | -.10 |
| Positive extreme video | Positive nuanced video | -0.29 | 0.31 | -0.84 | .999 | -.04 |
|  | Control  (no video) | -1.37 | 0.30 | -4.57 | <.001 | -.19 |
| Positive nuanced video | Control  (no video) | -1.11 | 0.30 | -3.70 | .002 | -.16 |

*Note*. *p*-value adjusted for comparing a family of 10.

**Table 2**

*Summary of results from Study 2*

|  | Unrealistic optimism bias | | | |  | | |
| --- | --- | --- | --- | --- | --- | --- | --- |
|  | Me | | My peer | |  |  |  |
| *Experimental condition* | *M* | *SD* | *M* | *SD* | *t* | *p_bonf_* | Cohen's *d* |
| Control (no video) | 6.43 | 2.38 | 7.14 | 2.32 | -5.52 | <.001 | -.23 |
| Positive extreme video | 5.07 | 1.93 | 5.78 | 2.27 | -5.20 | <.001 | -.22 |
| Positive nuanced video | 5.29 | 2.45 | 6.06 | 2.67 | -5.60 | <.001 | -.23 |
| Negative extreme video | 6.20 | 2.43 | 6.18 | 2.40 | 0.20 | .999 | .01 |
| Negative nuanced video | 6.00 | 2.43 | 6.15 | 2.34 | -1.17 | .999 | -.05 |

*Note*. *p*-value adjusted for comparing a family of 45.
