# Supplementary material for: Media intervention program for reducing unrealistic optimism bias: The link between unrealistic optimism, well‐being, and health
Source: Appl Psychol Health Well Being. 2021 Oct 24;14(2):499–518. doi: 10.1111/aphw.12316 (PMC9298214; doi:10.1111/aphw.12316)
Supplement: Supplementary file 4 — Table S1. Summary of results from Study 2 [file APHW-14-499-s001.docx]

**Table**

*Summary of results from Study 2*

|  | Unrealistic optimism bias | | | |  | | |
| --- | --- | --- | --- | --- | --- | --- | --- |
|  | Me | | My peer | |  |  |  |
| *Experimental condition* | *M* | *SD* | *M* | *SD* | *t* | *p_bonf_* | Cohen's *d* |
| Control (no video) | 6.43 | 2.38 | 7.14 | 2.32 | -5.52 | <.001 | -.23 |
| Negative video | 6.09 | 2.43 | 6.16 | 2.37 | -0.67 | .999 | -.03 |
| Positive video | 5.18 | 2.19 | 5.91 | 2.45 | -7.63 | <.001 | -.31 |

*Note*. *p*-value adjusted for comparing a family of 15.
