# Supplementary material for: Media intervention program for reducing unrealistic optimism bias: The link between unrealistic optimism, well‐being, and health
Source: Appl Psychol Health Well Being. 2021 Oct 24;14(2):499–518. doi: 10.1111/aphw.12316 (PMC9298214; doi:10.1111/aphw.12316)
Supplement: Supplementary file 5 — Table S1. Summary of the results from Study 3: Unrealistic optimism bias in all experimental conditions [file APHW-14-499-s002.docx]

**Table**

*Summary of the results from Study 3: Unrealistic optimism bias in all experimental conditions*

|  | Unrealistic optimism bias | | | |  | | |
| --- | --- | --- | --- | --- | --- | --- | --- |
|  | Me | | My peer | |  |  |  |
| *Experimental condition* | *M* | *SD* | *M* | *SD* | *t* | *p_bonf_* | Cohen's *d* |
| Article  positive |  |  |  |  |  |  |  |
| small group | 7.25 | 2.24 | 7.40 | 2.36 | -0.99 | .999 | -.03 |
| population | 6.89 | 2.24 | 7.30 | 2.14 | -2.68 | .999 | -.09 |
| Article  negative |  |  |  |  |  |  |  |
| small group | 7.40 | 2.20 | 7.39 | 2.13 | 0.06 | .999 | .01 |
| population | 7.20 | 1.90 | 7.78 | 2.03 | -3.8 | .023 | -.13 |
| Video  positive |  |  |  |  |  |  |  |
| small group | 7.53 | 2.23 | 8.13 | 2.06 | -3.98 | .012 | -.13 |
| population | 7.40 | 2.04 | 8.03 | 1.93 | -3.84 | .020 | -.13 |
| Video  negative |  |  |  |  |  |  |  |
| small group | 7.02 | 2.18 | 7.14 | 2.09 | -0.72 | .999 | -.02 |
| population | 7.16 | 2.31 | 7.41 | 2.23 | -1.53 | .999 | -.05 |
| Control (no media) | 7.03 | 2.33 | 7.63 | 2.10 | -3.95 | .013 | -.13 |

*Note*. *p*-value adjusted for comparing a family of 153.
