# Supplementary material for: Media intervention program for reducing unrealistic optimism bias: The link between unrealistic optimism, well‐being, and health
Source: Appl Psychol Health Well Being. 2021 Oct 24;14(2):499–518. doi: 10.1111/aphw.12316 (PMC9298214; doi:10.1111/aphw.12316)
Supplement: Supplementary file 6 — Table S1. Within‐subjects effects from Study 3: Unrealistic optimism bias assessment, behavior towards recommendations, and media type Table S2. Between‐subjects effects from Study 3: Behavior towards recommendations and media type [file APHW-14-499-s004.docx]

**Table**

*Within-subjects effects from Study 3: Unrealistic optimism bias assessment, behavior towards recommendations, and media type*

| *Cases* | *df* | *F* | *p* | η²_p_ |
| --- | --- | --- | --- | --- |
| Unrealistic bias assessment | 1 | 41.96 | <.001 | 0.05 |
| Unrealistic bias assessment*  behavior toward recommendations | 1 | 4.11 | .043 | >.0 |
| Unrealistic bias assessment*  media type | 1 | 1.15 | .283 | >.0 |
| Unrealistic bias assessment*  behavior toward recommendations*  media type | 1 | 4.35 | .037 | >.0 |
| Residuals | 802 |  |  |  |

**Table**

*Between-subjects effects from Study 3: Behavior towards recommendations and media type*

| *Cases* | *df* | *F* | *p* | η²_p_ |
| --- | --- | --- | --- | --- |
| Behavior toward recommendations | 1 | 1.61 | .205 | >.0 |
| Media type | 1 | 1.17 | .167 | >.0 |
| Behavior toward recommendations*  media type | 1 | 8.67 | .003 | .01 |
| Residuals | 802 |  |  |  |
