# Supplementary material for: Media intervention program for reducing unrealistic optimism bias: The link between unrealistic optimism, well‐being, and health
Source: Appl Psychol Health Well Being. 2021 Oct 24;14(2):499–518. doi: 10.1111/aphw.12316 (PMC9298214; doi:10.1111/aphw.12316)
Supplement: Supplementary file 7 — Table S1. Within‐subjects effects from Study 3: Unrealistic optimism bias assessment, behavior towards recommendations, and context Table S2. Between‐subjects effects from Study 3: Behavior toward recommendations and context [file APHW-14-499-s006.docx]

**Table**

*Within-subjects effects from Study 3: Unrealistic optimism bias assessment, behavior towards recommendations, and context*

| *Cases* | *df* | *F* | *p* | η²_p_ |
| --- | --- | --- | --- | --- |
| Unrealistic bias assessment | 1 | 42.43 | <.001 | 0.05 |
| Unrealistic bias assessment*  behavior toward recommendations | 1 | 3.91 | .048 | >.0 |
| Unrealistic bias assessment*  context | 1 | 5.92 | .015 | >.0 |
| Unrealistic bias assessment*  behavior toward recommendations*  context | 1 | 1.33 | .249 | >.0 |
| Residuals | 802 |  |  |  |

**Table**

*Between-subjects effects from Study 3: Behavior toward recommendations and context*

| *Cases* | *df* | *F* | *p* | η²_p_ |
| --- | --- | --- | --- | --- |
| Behavior toward recommendations | 1 | 1.26 | .262 | >.0 |
| context | 1 | 0.03 | .857 | >.0 |
| Behavior toward recommendations*  context | 1 | 1.53 | .216 | >.0 |
| Residuals | 802 |  |  |  |
